# Cardiac Rhythm and Molecular Docking Studies of Ion Channel Ligands with Cardiotoxicity in Zebrafish

**DOI:** 10.3390/cells8060566

**Published:** 2019-06-10

**Authors:** Bonifasius Putera Sampurna, Fiorency Santoso, Jia-Hau Lee, Wen-Hao Yu, Chin-Chung Wu, Gilbert Audira, Stevhen Juniardi, Jung-Ren Chen, Ying-Ting Lin, Chung-Der Hsiao

**Affiliations:** 1 Department of Bioscience Technology, Chung Yuan Christian University, Chung-Li 32023, Taiwan; boni_bt123@hotmail.com (B.P.S.); fiorency_santoso@yahoo.co.id (F.S.); gilbertaudira@yahoo.com (G.A.); stvn.jun@gmail.com (S.J.); 2 Department of Biotechnology, College of Life Science, Kaohsiung Medical University, Kaohsiung 80708, Taiwan; u105831002@kmu.edu.tw (J.-H.L.); u104022019@kmu.edu.tw (W.-H.Y.); 3 Graduate Institute of Natural Products, College of Pharmacy, Kaohsiung Medical University, Kaohsiung 80708, Taiwan; ccwu@kmu.edu.tw; 4 Department of Chemistry, Chung Yuan Christian University, Chung-Li 32023, Taiwan; 5 Department of Biological Science & Technology College of Medicine, I-Shou University, Kaohsiung 82445, Taiwan; jrchen@isu.edu.tw; 6 Center of Nanotechnology, Chung Yuan Christian University, Chung-Li 32023, Taiwan; 7 Center of Biomedical Technology, Chung Yuan Christian University, Chung-Li 32023, Taiwan

**Keywords:** zebrafish, heart, ion channel ligand, cardiotoxicity, molecular docking

## Abstract

Safety is one of the most important and critical issues in drug development. Many drugs were abandoned in clinical trials and retracted from the market because of unknown side effects. Cardiotoxicity is one of the most common reasons for drug retraction due to its potential side effects, i.e., inducing either tachycardia, bradycardia or arrhythmia. The zebrafish model could be used to screen drug libraries with potential cardiotoxicity in a high-throughput manner. In addition, the fundamental principles of replacement, reduction, and refinement of laboratory animal usage, 3R, could be achieved by using zebrafish as an alternative to animal models. In this study, we used a simple ImageJ-based method to evaluate and screen 70 ion channel ligands and successfully identify six compounds with strong cardiotoxicity in vivo. Next, we conducted an in silico-based molecular docking simulation to elucidate five identified compounds that might interact with domain III or domain IV of the *Danio rerio* L-type calcium channel (LTCC), a known pharmaceutically important target for arrhythmia. In conclusion, in this study, we provide a web lab and dry lab combinatorial approach to perform in vivo cardiotoxicity drug screening and in silico mechanistic studies.

## 1. Introduction

### 1.1. Alterations in Cardiac Structure and Function by Drugs

Drugs can cause side effects. The safety of the drugs is a crucial aspect in the drug development and control. Cardiotoxicity is one of the most common reasons for drug retraction from the market. Safety labels have been revised to give adequate warning about potential side effects. Many drugs have also been abandoned from the clinical trials because of unanticipated toxic effects [1]. Many countries, including the USA and France, revised and tightened regulations on new compounds. For example, Benfluorex had been in the market since 1976 and was withdrawn in 2009, because its risks, particularly on heart valve disease (fenfluramine-like cardiovascular side effects), were greater than its benefits. The “Inspection Générale des Affaires Sociales” (IGAS) is the French Government audit, evaluation and inspection office for health, social security, social cohesion, employment and labour policies and organisations. The IGAS of France investigated the Benfluorex case to determine the mechanism, sequence of events, and decision making of drug regulation. Both fenfluramine and benfluorex were found to form norfenfluramine as a metabolite. Such a side effect led to the withdrawal of fenfluramine as an anorectic drug worldwide, and later, to the withdrawal of benfluorex in Europe [2]. Arrhythmia can be caused by many factors, such as genetical mutations, environmental factors, and drugs that alter heart repolarization. Cardiotoxicity is of great concern to the pharmaceutical industry, regulatory agencies, and society, as it has a huge socio-economic impact [3].

### 1.2. Zebrafish as Cardiotoxicity Animal Model

Assessments of the drug cardiotoxicity usually can be done in vitro by cardiomyocyte culture [4] or in vivo with animal models [5]. Drug assessments using animal models are usually done before clinical trials, but can also be done afterwards to verify the side effect(s) [6]. Cell-based studies provides high-throughput screening but lack relevant whole organism physiology. Mammalian models provide verification of in vivo data, but are not suited for high-throughput screening. Zebrafish is an emerging animal model gaining global importance in biomedical research. In the early stage, zebrafish provides several advantages, such as a high number of offspring, ex vivo fertilization, and unsurpassed optical clarity. Zebrafish embryos have been approved as an excellent alternative animal model for high-throughput screening [7]. The unique advantages of zebrafish could further strengthened its position as an ideal model for drug toxicity screening that bridges the gap between in vitro and in vivo cell-based studies. Zebrafish, as a low vertebrate, have organs; in particular, their heart serves as a useful cardiotoxicity model [8].

In the early stages, zebrafish provide the advantages of optically transparency and development outside of the mother, making functional and morphological analysis possible without invasive measures. Melanophores start to appear in zebrafish 24 h post fertilization (hpf) on either side of the head and continuing down the trunk, followed by iridophores. Without a pigment inhibition procedure, the pericardium area pigmented after 120 hpf and heart beat analysis cannot be conducted [9]. One day after fertilization, somitogenesis is complete and many organ rudiments have been laid down [10]. One important aspect that has received little attention is the ability of zebrafish larvae to metabolize drugs over time [11]. Cardiac progenitors emerged after 14 hpf. Cardiac precursors migrate and fuse at the midline by 19 hpf. One day after fertilization, the beating linear heart tube has formed and circulation begins. The cardiac chamber is formed and differentiated two days after fertilization [12]. The zebrafish heart consists of two contractile chambers—the atrium and the ventricle—delimited by one valve on the atrioventricular junction. Mammals including humans have four chambers and a double circuit circulation system, while fish only have a single circuit circulation system [13]. Although the number of chambers and circuit circulation system is different, zebrafish and human hearts share some similarities, e.g., basic contractile dynamics and action potential morphology [14]. Zebrafish action potential (AP) shares the main characteristic of human cardiac AP. The ventricle AP of zebrafish heart has a long plateau, similar to the human cardiac AP, and consequently, a distinct QT-interval in the Electrocardiogram (ECG) [15]. All phases (0-4) of AP in human heart are also present in the zebrafish heart (except the rapid phase-1 repolarization). This means a tiny transient outward current (I_TO_), or an absence thereof, in zebrafish heart [16]. Depolarization originated from the sinoatrial (SA) region, as determined by voltage dynamic visualization. This location is considered to be the same as the human sinoatrial node (SAN) [17].

### 1.3. Evaluation of Cardiovascular Toxicity Mechanism by In Silico Molecular Docking

**Figure 1:**
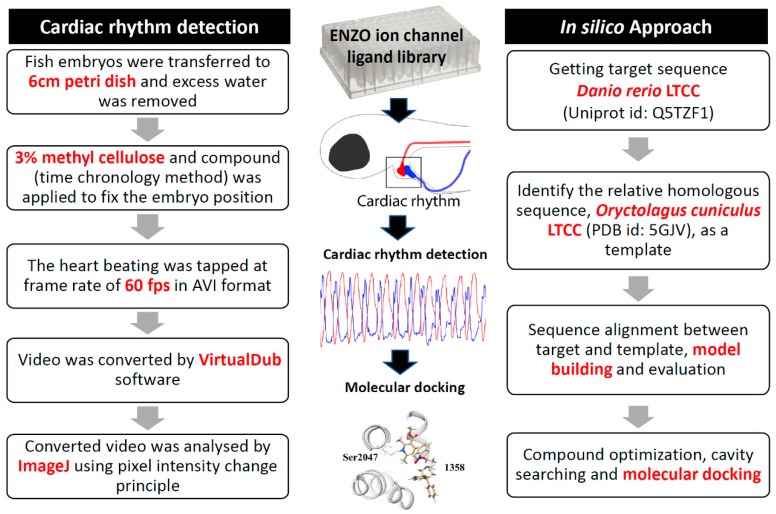
Overview of experimental design and workflow used in this study. We adapted the web lab approach to perform ion channel ligand library in vivo screen based on cardiac rhythm regularity in zebrafish embryos (**left** panel). Once the candidate compounds had been identified, in silico molecular docking was performed to study the ligand and its target protein interaction (**right** panel).

Structure-based molecular simulation provides an excellent and powerful tool to elucidate a potential ligand-target protein binding mechanism. Thanks to great improvements in computational capacity and efficiency, molecular docking has, in recent years, provided a powerful and popular tool to elucidate the molecular interaction between the ligand and its binding protein at atomic resolution [18,19,20,21,22]. This approach is especially important for the pharmaceutical industry for lead drug development/improvement or drug toxicity predictions. The outcomes of in silico molecular docking can also provide guidelines on modifying lead drugs and designing new compounds with superior binding affinities [23,24]. In a previous publication, we established a simple image-based method using the ImageJ as the main platform to analyse the heartbeat rhythm in zebrafish embryos [25]. In this study, we conducted image-based cardiac rhythm measurements to perform wet lab ion channel ligand library screening to explore potential chemicals carrying cardiovascular toxicity. Once candidates were identified, a dry lab molecular docking approach was utilized to provide more solid evidence supporting their ligand-protein interactions at an atomic scale. To gain a 3D protein structure, we first retrieved the target sequence from UniProt (https://www.uniprot.org/), and then identified the relative homologous structure as a template. By doing the sequence alignment between the target and template, we could build up 3D protein structure models to see whether the identified compounds could bind to the best target protein. We also optimized the compound structure, searched for potential binding cavities, and conducted molecular docking for the ligand-protein interaction. This in vivo and in silico combinational approach for cardiotoxicity screen is illustrated in Figure 1.

## 2. Material and Methods

### 2.1. Zebrafish Maintenance and Embryo Collection

Wild type zebrafish were raised according to the procedure described in the literature [26]. Pairs were setup in the evening of the day before collection. In the morning, embryos were collected and kept in an incubator at 28 °C. Seventy-two hpf larvae were used for this study. The animal protocol was approved by the Institutional Animal Care and Use Committee of Chung Yuan Christian University, with approval number 106025 (25 May 2017).

### 2.2. Video Recording, Processing, and Analysis

The whole procedure of video recording, image processing and data analysis for cardiac rhythms was described in our previously published ImageJ-based protocol [25].

### 2.3. Drug Treatment

The Screen-Well ion channel ligand library (BML-2805, Enzo, Farmingdale, NY, USA) was used to perform cardiotoxicity screening. A total of 70 compounds are included in the library: 7 intracellular calcium, 24 calcium, 23 potassium, 10 sodium, and 6 miscellaneous channel openers or blockers. Stock concentrations for each compound in the library are 10 mM with 100 µL volume. The compounds are diluted with distilled water to make working solutions at 10 µM. For drug treatment, 20 µM of the compound stock was dropped onto the fish mounted with the same volume (around 50 µL) of 3% methylcellulose for a final working concentration of 10 µM. The heart beats from three zebrafish larvae were used for the first round of screening. The compounds that exceeded normal ranges, displaying either bradycardia, tachycardia or total heart stop, were then subjected to a detailed chronological examination. The heart beats from five zebrafish larvae for each compound were recorded for 10 sec at 5 min intervals over a period of 30 min.

### 2.4. Statistical Analysis

Statistical and graphic analyses were done using the GraphPad Prism 7 software (Available online: https://www.graphpad.com/scientific-software/prism/). A column table option was used to enter the data and replicate values, stacked into columns. Data in the endpoint and chronological methods were presented as mean ± SEM, and a t-test (with parametric assay) was used to calculate the significance. An unpaired student t-test was applied to compare the significance within two groups. Sample distribution was assumed to be normally distributed. A difference between two means was considered significant when * *p* < 0.05, ** *p* < 0.005, *** *p* < 0.001 and **** *p* < 0.0001.

### 2.5. Structure-Based Molecular Simulation for the Binding of Selected Ligands with Danio Rerio LTCC

To investigate the interactions between the identified ligands with Danio rerio LTCC, we performed a structure-based molecular simulation study comprising homology modeling and molecular docking on an Asus personal computer with Intel(R) Core(TM) i7 2.67 GHz processor, running Windows 7, using Modeller Software v9.20 [27] and Discovery Studio 3.0 (DS 3.0; Discovery Studio Modeling Environment, Accelrys Software Inc, San Diego, 2005–2010) [28]. To build a 3-dimensional model for Danio rerio LTCC by homology modeling, we used a specific amino acid sequence (UniProt id: Q5TZF1) (Cav1.2) to locate homologous protein sequences in the Protein Data Bank (PDB) database. The NCBI blastp (https://blast.ncbi.nlm.nih.gov/Blast.cgi) tool was used in the target search. As a result, the LTCC structure of European rabbit (Oryctolagus cuniculus) (PDB id: 5GJV) was identified as the best template structure candidate for homology modeling. The best of the ten generated models was chosen by the three best scores of molpdf, DOPE [29], and GA341 (Modeller) [30]. Multiple homology models were automatically generated by the software, Modeller. DOPE refers to “Discrete Optimized Protein Energy”, a statistical potential optimized for model assessments. When DOPE is lower, the model is more stable. GA341 uses the percentage sequence identity between the template and the model as a parameter. GA341 scores always range from 0.0 (worst) to 1.0 (native-like).

To see whether the identified compounds could bind to Danio rerio LTCC, we also conducted docking simulation studies using the docking module in DS3.0, LigandFit [31]. A cavity searching method, the eraser algorithm [32], located two possible binding pockets in domains III and IV of the built 3D model of the Cav1.2 transmembrane proteins. As mentioned, the two cavities located in DIII and DIV have significant pharmaceutical potential. The five compounds, 1345, 1346, 1358, 1360, 1377, were then optimized (Chem3D) using the mmff94 forcefield until the structural energy converged to the root-mean-square-deviation gradient within 0.05 kcal mol-1 Å-1. The receptor hydrogens automatically restored, and all the atoms’ partial charges were assigned using the parameters of the CHARMm force field [33]. The scoring function of Dock measured the interaction energy between the ligand pose and receptor. Any docking pose fitting into the shape of the generated cavity was required. A higher score indicated a stronger binding affinity. In summary, the best poses of 5 compounds after docking were visualized in the UCSF Chimera 1.13 graphics environment [34].

## 3. Results

### 3.1. Overview of Experimental Design and Workflow

In this study, the potential cardiac rhythm alteration in zebrafish after exposure to compounds in an ion channel ligand library was conducted; the workflow is summarized in the left panel of Figure 1. For the cardiac rhythm assay, we mounted zebrafish embryos aged at 72 hpf with 3% methylcellulose to reduce their movement, and later applied chemicals from an ion channel ligand library (containing 70 compounds) at 10 μM to measure the changes in cardiac rhythm according to our previous ImageJ-based method [25]. In order to prevent the potential side effects of anesthesia on heart rate or rhythmicity, we used methylcellulose to mount zebrafish larvae for videotaping. Methylcellulose is not digestible, toxic, or allergenic. The basic principle of our ImageJ-based method is to measure the pixel changes of the blood cells in the atrium and ventricle during the muscle contraction and relaxation phases. This method makes it possible to perform functional assessments on the cardiovascular toxicity of compounds in zebrafish embryos based on video images. Once the candidate compounds were identified, we conducted in silico molecular docking to provide more solid evidence to provide evidence for ligand-binding protein interactions at an atomic level (right panel, Figure 1).

### 3.2. Evaluation of Chemical Cardiotoxicity by End-Point Method

**Figure 2:**
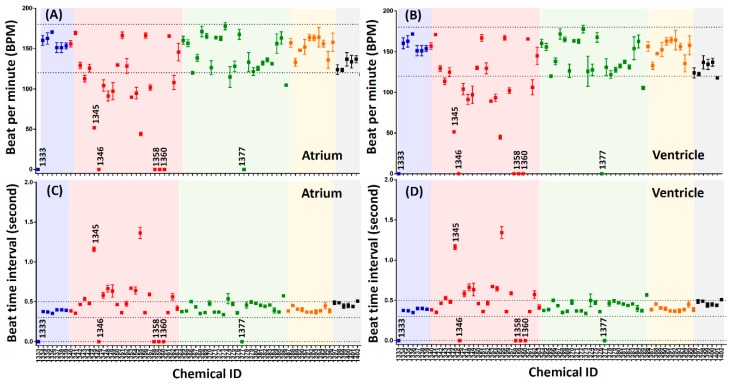
The end-point screen result for ion channel ligand library by using zebrafish embryos aged 72 hpf. (**A**) Atrium and (**B**) Ventricle beat per minute (BPM) for each compound screened. (**C**) Atrium and (**D**) Ventricle beat time interval (rhythmicity) for each compound screened. The color code is blue for intracellular calcium channels, red for calcium channels, green for potassium channels, yellow for sodium channels, and grey for miscellaneous channels. The numbers above some points shows the candidate compounds which were shown to induce irregular heart rhythmicity by using end point screening method. They are compound 1333 (Antibiotic A-23187), 1345 (Flunarizine hydrochloride), 1346 (FPL-64176), 1358 (Niguldipine-HCl), 1360 (Pimozide) and 1377 (Fluspirilene), respectively.

Initially, we used an image-based end-point method to screen the potential effects of an ion channel ligand library on heart beat rhythm alterations. This ion channel ligand library contains 70 ion channel blockers and openers for characterizing and identifying ion channels in individual cells or tissue. This ion channel ligand library covers diverse ion channel blockers or activators (7 intracellular calcium channels, 24 calcium channels, 23 potassium channels, 10 sodium channels, and 6 miscellaneous channels) that might affect heart depolarization and repolarization processes. In order to screen a potential compound with high cardiotoxicity at a low dose, we performed chemical screening at 10 μM as a starting point. The normal zebrafish aged 72 h post-fertilization (hpf) has a heartbeat interval of around 0.3–0.5 s and a rate of around 120–180 beat per minute (bpm) [35]. A heartbeat that exceeds these ranges is considered abnormal. The ion channel ligand library screen result shows that zebrafish embryos aged at 72 hpf display either bradycardia, tachycardia or total heart stop after treatment with some ion channel library compounds. Five compounds (1333, 1346, 1358, 1360 and 1377) exhibiting ventricular fibrillation or total heart stop in first step screening (end-point screen) were identified. Compounds number: 1343, 1345, 1347, 1348, 1349, 1353, 1354, 1355, 1357, 1362, 1366, 1386 showed a slight slowing of the heartbeat (bradycardia). Two compounds (1338 and 1373) could induced a rate of more than 180 bpm, indicating that these compounds have a tachycardia-inducing potential (Figure 2).

### 3.3. Validation of Primary Screen Data by Chronology Method

In our first-round end-point screening, atrioventricular block could not be detected, and we hypothesized that the A-V blocking effect might take place soon after the compound was administered. A chronological video recording method is necessary to verify the result and observe the severity of each compound. To achieve this goal, the 16 compounds that passed the first-round screening by the end-point method were subjected to a detail chronological examination. After drug administration, we video recorded the heart beat for 10 sec at 5 min intervals, over a period of 30 min.

**Figure 3:**
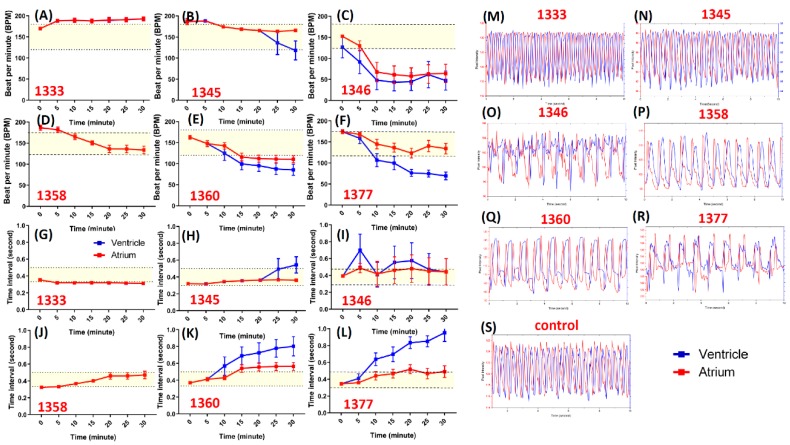
The chronology screen result for ion channel ligand library by using zebrafish embryos aged 72 hpf. (**A**) Atrium (red color) and ventricle (blue color) beat per minute (BPM) and (**G**) beat time interval for compound 1333 screened. (**B**) BPM and (**H**) Beat time interval for compound 1345 screened. (**C**) BPM and (**I**) Beat time interval for compound 1346 screened. (**D**) BPM and (**J**) Beat time interval for compound 1358 screened. (**E**) BPM and (**K**) Beat time interval for compound 1360 screened. (**F**) BPM and (**L**) Beat time interval for compound 1377. (**M**–**S**) Atrium and ventricle rhythmicity pattern after 30 min incubation of compounds number 1333 (Antibiotic A-23187), 1345 (Flunarizine hydrochloride), 1346 (FPL-64176), 1358 (Niguldipine-HCl), 1360 (Pimozide), 1377 (Fluspirilene), and control.

The results showed that six compounds displayed typical arrhythmia phenotypes with different severities (Figure 3). For example, compound 1333 (Antibiotic A-23187) caused a tachycardia effect from 5 min onwards after drug treatment (Figure 3A,G), compared to a bradycardia effect in the first step end-point screening. Atrium and ventricle pattern after 30 minutes’ incubation showed 1:1 rhythm, but the peak was more compact compared (Figure 3M) to the control (Figure 3S). Compound 1345 (Flunarizine hydrochloride) caused a slowing of heart rate as well as arrhythmia after incubation for 25 and 30 min (Figure 3B,H). After 30 min of incubation, arryhtmia was not shown in the pattern; however, the peak was less compact compared to the control (Figure 3N). Compound 1346 (FPL-64176) caused arrhythmia (Figure 3C,I). Based on the pattern, after 30 min of incubation, the atrium and ventricle showed arrhythmic beating (Figure 3O). Compound 1358 (Niguldipine-HCl) caused bradycardia effect after 10 min of treatment, even though the time interval and heart rates (bpm) did not exceed the normal boundaries (Figure 3D,J). The beating rhythm between the atrium and ventricle had a 1:1 rhythm, but the heart slowed nonetheless (Figure 3P). Compound 1360 (Pimozide) induced atrioventricular block from 10 min onwards (Figure 3E,K). After 30 min of incubation, the atrium and ventricle became arrhythmic, with one peak in the ventricle being followed by two in the atrium (Figure 3Q). Compound 1377 (Fluspirilene) induced arrhythmia from 5 min onwards after drug treatment (Figure 3F,L) compared to the first-step endpoint screening that already showed total heart stop. This is in line with the atrium and ventricle peak that became arrhythmic, with one peak of the ventricle being followed by two of the atrium (Figure 3R).Therefore, this study suggests that the atrioventricular block caused by the compound could be studied in detail from using the time chronology method.

**Figure A1:**
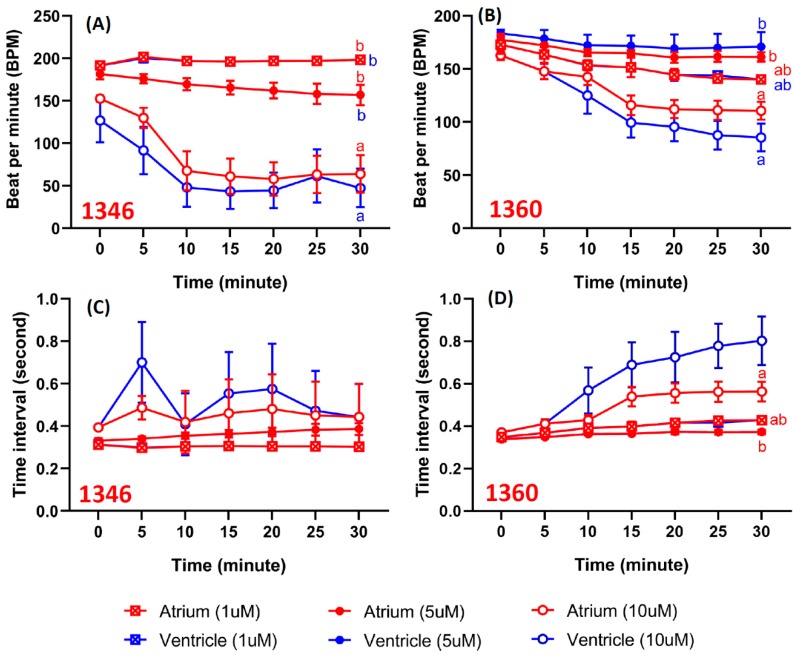
Dose response experiment to show the lowest effective dose (LOED) of compound to induce heart rate alteration and rhythmicity dysregulation. The time chronology change of heart beat rate (red color for atrium and blue color for ventricle) after compound 1346 (FPL-64176) and 1360 (Pimozide) treatment was shown in **A** and **B**. The time chronology of heart rhythmicity (red color for atrium and blue color for ventricle) after compound 1346 and 1360 treatment was shown in **C** and **D**. The data was expressed as mean ± S.E.M. and bars sharing a letter are not significantly different from one another as assessed by one-way ANOVA, followed by Fisher’s least significant difference test (p < 0.05). Five zebrafish larvae aged at 72 h post-fertilization (hpf) were used in this assay.

In addition, two compounds, i.e., 1346 (FPL-64176) and 1360 (Pimozide), that induce severe cardiotoxicity (arrythymia) were selected to test the lowest effective dose (LOED). Three different doses, 1, 5 and 10 μM, of the tested compounds were given to zebrafish embryos aged 72 hpf, and the heart beat rate and rhythmicity were recorded for comparison by the time chronology method. As shown in Figure A1, a low concentration (1 µM and 5 µM) in compound 1346 or 1360 did not change the heart beat rate (Figure A1A,B) and cardiac rhythmicity (Figure A1C,D) of zebrafish larvae. From tests with those two compounds, it can be concluded that the cardiotoxicity effect for our tested ion channel ligands was largely located around the 10 μM range.

**Figure A2:**
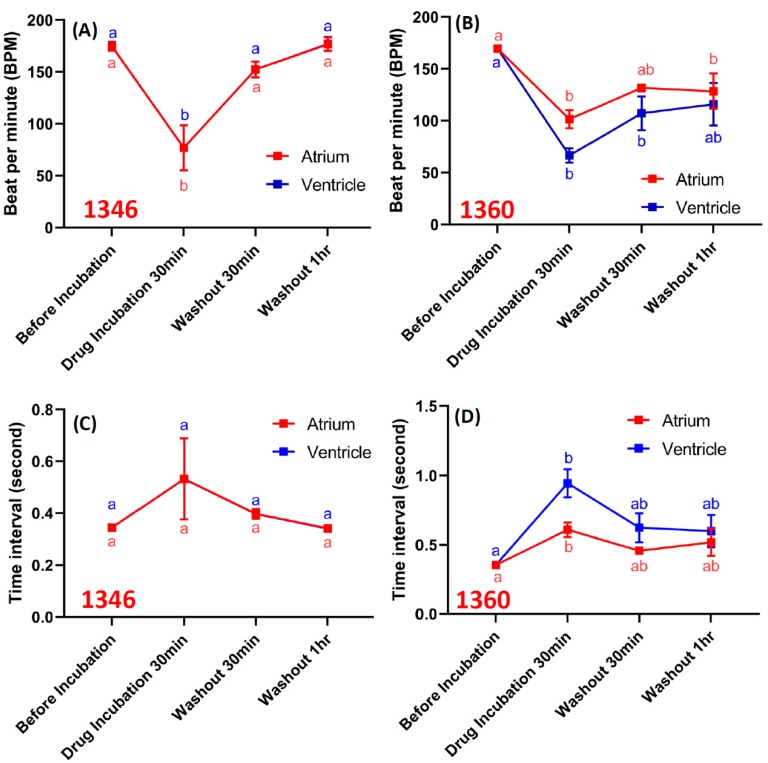
Washout experiment to show the heart rate and rhythmicity can be restored in the absence of the compound. The heart beat rate (red color for artrium and blue color for ventricle) before, during and after compound 1346 (FPL-64176) and 1360 (Pimozide) treatment was shown in **A** and **B**. The heart rhythmicity (red color for artrium and blue color for ventricle) before, during and after compound 1346 and 1360 treatment was shown in **C** and **D**. The data was expressed as mean ± S.E.M. and bars sharing a letter are not significantly different from one another as assessed by one-way ANOVA, followed by Fisher’s least significant difference test (*p* < 0.05). Five zebrafish larvae aged at 72 h post-fertilization (hpf) were used in this assay.

Finally, we wondered whether the cardiotoxicity induced by ion channel ligands could be restored once those compounds were removed. We addressed this question by a washout approach for compounds 1346 (FPL-64176) and 1360 (Pimozide), both of which induce severe cardiotoxicity (arrythymia). Based on Figure A2, we conclude that for compound 1346, washout experiments for 30 min and 1 h could restore the heart rate to the normal range (Figure A2A,C). On the other hand, for compound 1360, a washout experiment for 30 min and 1 h may restore the heart rate to some extent, but still to withing the normal boundaries (Figure A2B). The washout experiments were also unable to fully reverse the arrhythmic effect of compound 1360 on zebrafish larvae aged 72 hpf (Figure A2C). It can be concluded that LOED and washout rescue experiments are useful methods to double validate the serveity of compound cardiotoxicity. It takes more dilution fold to reach the LOED, and more time to fully return to normal heart beat levels and rhythmicity if the tested compound shows elevated cardiotoxicity.

### 3.4. Molecular Docking Using the Danio Rerio Cav1.2 Homology Modeling Structure

For molecular docking, the candidate protein we selected was *Danio rerio* L-type calcium channel (LTCC). LTCC contains four subtypes proteins (Cav1.1 to 1.4) and plays important roles in passing inward Ca^2+^ current and triggering calcium release from the sarcoplasmic reticulum by activating ryanodine receptor 2 (RyR2). The permeability to calcium and contractility of LTCC can be tightly controlled by protein phosphorylation. Much research has shown that molecules which specifically modulate the LTCC Ca^2+^ current bind to a particular area of the Cav1.2. domain III (DIII) or domain IV (DIV) [36,37,38]. The expression of Cav1.2 is known to be rich in cardiac and vascular smooth muscle, and thus, it may be considered pharmaceutically important [39,40].

Our simulation studies contain three computational methods: homology modeling, cavity searching, and molecular docking. Since the *Danio rerio* Cav1.2 protein crystal structure is not available, we built a 3D structure model through homology modelling by using its mammalian version from European rabbit (*Oryctolagus cuniculus*). The two cavities were discovered in DIII and DIV of the best *Danio rerio* Cav1.2 model. The sequence similarity between zebrafish and European rabbit in DIII and DIV was around 81.6% and 78.5%, respectively. We then docked the five identified compounds—with the exception of compound 1333—to these two binding pockets. Compound 1333 (Antibiotic A23187) is an ion-carrier, which can induce an influx of calcium to increase the LTCC concentration gradient [41].

**Figure 4:**
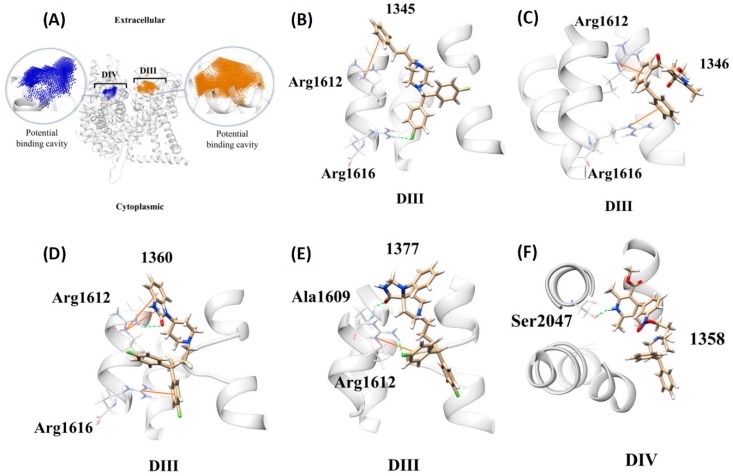
The molecular docking poses of compounds 1345, 1346, 1358, 1360 and 1377 based on the homology modeling with *Danio rerio* Cav1.2 protein sequence. (**A**) The two located cavities in domain III (DIII) and IV (DIV) of the modeling Cav1.2 are colored blue and orange, respectively. The best docking poses for compounds 1345 (Flunarizine hydrochloride, **B**), 1346 (FPL-64176, **C**), 1360 (Pimozide, **D**), and 1377 (Fluspirilene, **E**) are in the DIII cavity. However, the best pose for compound 1358 (Niguldipine-HCl, **F**) is in the DIV cavity. Note that the hydrogen bonds are represented in green dotted lines while π-cation interactions are in orange lines. The carbon atoms of proteins are colored white. The carbon atoms of the best docking poses are colored tan.

To investigate whether another five identified ligands could bind with *Danio rerio* LTCC, we constructed the homology protein structure using the software Modeller and created binding cavities from the homology model by the erase algorithm implemented in the Discovery Studio software, and identified two putative pharmaceutical cavities, as shown in Figure 4A (highlighted in blue and orange). In Figure 4B, compound 1345 (Flunarizine hydrochloride) formed one π-cation interaction with DIII Arg1612 and hydrogen bond with DIII Arg1616. Compound 1346 (FPL-64176) formed π-cation interactions with DIII Arg1612 and DIII Arg1616 (Figure 4C). Also, compound 1360 (Pimozide) formed one hydrogen bond and two π-cation interactions with DIII Arg1612 and one π-cation interaction with DIII Arg1616 (Figure 4D). Next, compound 1377 (Fluspirilene) formed one hydrogen bond with DIII Ala1609 and one hydrogen bond and π-cation interaction with DIII Arg1612 (Figure 4E). By contrast, compound 1358 (Niguldipine-HCl) formed one hydrogen bond with DIV Ser2047 (Figure 4F). In conclusion, the docking results showed that the five identified compounds may bind to the domain III or IV of the *Danio rerio* LTCC to interfere with its function, thereby possibly leading to arrhythmia.

## 4. Discussion

This paper described a simple and efficient pipeline for in vivo and in silico cardiotoxicity screening in zebrafish using an image-based and molecular docking methodology. The advantages of this pipeline are that the potential cardiotoxic compound can be quickly screened using an image-based method to evaluate the atrium and ventricle beating rhythm. The early cardiotoxic effect can be validated using a chronology method. By using this approach, we were able to screen an ion channel ligand library containing 70 chemicals, and successfully identified several compounds that induce severe arrhythmia in zebrafish, and which therefore have high cardiotoxicity. To our knowledge, our protocol is the most simple to date for in vivo cardiotoxicity screening.

The previous cardiotoxicity evaluation method in zebrafish largely relied upon a 96 hr acute exposure protocol in which the chemicals of interest were exposed from 0 to 96 hpf. This 96 hr continuous exposure protocol suffered from a high noise problem, since heart abnormalities might be caused by potential side effects of the tested compound, i.e., on developmental retardation rather than its organ-specific toxicity. Recently, a new concept called ZeGlobalTox has been proposed that changed the exposure procedure after 96 hpf and checks the heart rate regularity immediately at 100 hpf [42]. Our current protocol is similar to ZeGlobalTox, but checks the heart beat regularity change in a more immediate fashion. The tested compound dose given in our test was much lower than those used in the previously published method, and its potential toxic effect was checked immediately after administration. This approach led us to identify several compounds with high cardiotoxicity which induced arrythmia in zebrafish.

In the first screening method, Antibiotic A-23187 caused ventricular fibrillation after the fish were incubated for 30 min. The time chronology method used to observe the severity of Antibiotic A-23187 showed the opposite result. Tachycardia occurred after five minutes’ incubation. Antibiotic A-23187 (calcimycin) is an antibiotic ionophore which transports divalent cations across the cell membrane into the cytoplasm and releases cations from intracellular storage sites [43]. The divalent cation ionophore A-23187 produces numerous biological effects in both myocardial and non-myocardial tissues or membrane preparations. Calcimycin enhances the force of contraction of ventricular, atrial, and myocardial tissues [44]. The ability of calcimycin to transport Ca^2+^ ions across membranes can yield direct or indirect positive inotropic effects (increase heart rate). The same result was observed in rats, where the addition of calcimycin increased the heart beat by up to 50% [45].

The Ca^2+^ channel blocker flunarizine, a lipophilic diphenylpiperazine derivative with calmodulin binding properties and histamine H1 blocking activity, is used in the prophylactic treatment of migraines, in the treatment of vertigo, and as an adjuvant in the treatment of epilepsy. Flunarizine inhibits the influx of extracellular calcium through myocardial and vascular membrane pores by physically plugging the channel. The decrease in intracellular calcium inhibits contractile processes of smooth muscle cells, causing dilation of the coronary and systemic arteries, increased oxygen delivery to the myocardial tissue, decreased total peripheral resistance, decreased systemic blood pressure, and decreased afterload [46]. In this study, compound 1345 (Flunarizine hydrochloride) caused a slowing of the heart as well as arrhythmia after incubation for 25 and 30 min. The same result was observed in another study which stated that Flunarizine induced arrhythmias in anaesthetised rats [47].

The endpoint method analysis of FPL-64176 showed that the heart completely stopped after 30 min incubation with FPL-64176. FPL-64176 is a specific L-type calcium channel modulator. This compound can prolong both the opening of L-type Ca^2+^ channels during depolarization and the time of inactivation during repolarization [48]. FPL-64176 might have some negative inotropic effects. Abnormalities in the Ca^2+^ homeostatic process could trigger arrhythmia, such as disorganized electrical activity in the ventricles associated with ventricular fibrillation leading to sudden cardiac death [49]. When cardiac cells are overloaded with Ca^2+^, the excessive number of ions leads to propagating Ca^2+^ waves. The waves would cause delayed afterdepolarization via Ca^2+^ activated inward current, leading into arrhythmia [50].

Niguldipine HCl is a L-type calcium channel blocker which can also inhibit T-type calcium channels at higher concentrations [51]. In the first screening, treated zebrafish embryo hearts stopped completely after incubation with niguldipine HCl. This result was verified with the time chronology method, where bradycardia occurred after 10 min of treatment. This result is in line with previous study, which found that calcium channel blockers showed a direct negative chronotropic effect [52].

Five minutes after pimozide incubation, atrioventricular block occurred, subsequently becoming more severe over time. This result is in line with previous study. Pimozide reportedly caused acquired long QT syndrome and ventricle arrhythmia [53]. The main purpose of Pimozide is as an antipsychotic drug to treat Tourrete’s syndrome and other psychiatric disorder. These cardiotoxicity effects may be associated with high dose intake or interactions with other medication that could slow the drug’s rate of metabolism [54]. Very low concentrations of the compound generate prolonged QT, suggesting a high affinity interaction with cardiac K^+^ channels. IC_50_ of hERG channel currents occurred at 18 nM, with significant inhibition as low as at 3 nM. The cardiotoxicity effect of Pimozide is poorly reversible, indicating an intracellular site of action and/or a high affinity interaction [53]. Our washout experiment also supported this point of view, since the slow down of heart beat could not be fully restored, even after 1 h of washout treatment (Figure A2B).

Fluspirilene is a Na^+^ and Ca^2+^ channel inhibitor. The activity of this compound is not selective for the Ca^2+^ ion channel, but is more closely associated with Na^+^ channel blockers [55]. In the first screening, treated zebrafish embryo heart completely stopped after incubation with fluspirilene. In the time chronology method, arrhythmia occurred from 5 min onwards and became more severe over time. This result is in line with a previous study that found Fluspirilene could cause atrioventricular block in zebrafish embryo heart [56].

It is worth mentioning that although the administration of methylcellulose as a mounting medium can prevent embryo movement, thereby facilitating videotaping, it might also reduce drug delivery efficiency. The intake of a drug via a methylcellulose vehicle may affect the efficiency of the drug; this has been reported previously [57]. This was proven by the different severity of the drug detected in the endpoint and time chronology methods by the same incubation time. For the end point method (without methycellulose administration), 30 min of incubation showed total heart stop, but only atrioventricular block in the chronology method (with methycellulose administration). Even though drug severity might differ between the two treatments, the progression of heart alteration occurs in the same manner. Fluspirilene as an example, based on previous study, could induce atrioventricular block (2:1); this situation was observed in the time chronology method from time to time, but not in the end-point method. Regardless, atrioventricular block might develop into total heart stop [56].

In addition to arrythymia functional assessement, we further conducted a structure-based molecular simulation on the homology modeling *Danio rerio* 3D structure to investigate whether the identified ligands could bind with *Danio rerio* LTCC. The eraser algorithm produces the two possible *Danio rerio* binding pockets in domains III and IV of the built 3D model of the Cav1.2 transmembrane proteins. Comparing to the recognized knowledge of literature as previously described, we consider these two binding sites to be of pharmaceutical significance. The results of molecular docking supported five arrhythmia-inducing compounds in zebrafish embryos, and might mediate by functional compromise of Cav1.2 LTCC through DIII or DIV blockage.
